# Anxiety onset in adolescents: a machine-learning prediction

**DOI:** 10.1038/s41380-022-01840-z

**Published:** 2022-12-08

**Authors:** Alice V. Chavanne, Marie Laure Paillère Martinot, Jani Penttilä, Yvonne Grimmer, Patricia Conrod, Argyris Stringaris, Betteke van Noort, Corinna Isensee, Andreas Becker, Tobias Banaschewski, Arun L. W. Bokde, Sylvane Desrivières, Herta Flor, Antoine Grigis, Hugh Garavan, Penny Gowland, Andreas Heinz, Rüdiger Brühl, Frauke Nees, Dimitri Papadopoulos Orfanos, Tomáš Paus, Luise Poustka, Sarah Hohmann, Sabina Millenet, Juliane H. Fröhner, Michael N. Smolka, Henrik Walter, Robert Whelan, Gunter Schumann, Jean-Luc Martinot, Eric Artiges, IMAGEN consortium, Eric Artiges, Semiha Aydin, Christine Bach, Tobias Banaschewski, Alexis Barbot, Gareth Barker, Arun Bokde, Nadège Bordas, Zuleima Bricaud, Uli Bromberg, Ruediger Bruehl, Christian Büchel, Anna Cattrell, Patricia Conrod, Sylvane Desrivieres, Tahmine Fadai, Irina Filippi, Herta Flor, Vincent Frouin, André Galinowski, Jürgen Gallinat, Hugh Garavan, Fanny Gollier Briand, Chantal Gourlan, Penny Gowland, Stella Guldner, Andreas Heinz, Bernd Ittermann, Tianye Jia, Hervé Lemaitre, Jean-Luc Martinot, Jessica Massicotte, Ruben Miranda, Kathrin Müller, Frauke Nees, Charlotte Nymberg, Marie Laure Paillère Martinot, Tomas Paus, Zdenka Pausova, Jean-Baptiste Poline, Luise Poustka, Jan Reuter, John Rogers, Barbara Ruggeri, Anna S. Sarvasmaa, Christine Schmäl, Gunter Schumann, Maren Struve, Michael Smolka, Wolfgang Sommer, Hélène Vulser, Henrik Walter, Robert Whelan

**Affiliations:** 1 Université Paris-Saclay, Institut National de la Santé et de la Recherche Médicale, INSERM U1299 “Trajectoires développementales Psychiatrie”, Ecole Normale Supérieure Paris-Saclay, CNRS UMR 9010, Centre Borelli, Gif-sur-Yvette, France; 2 Department of Psychology, Humboldt-Universität zu Berlin, Berlin, Germany; 3 Department of Child and Adolescent Psychiatry, Pitié-Salpêtrière Hospital, AP-HP, Sorbonne Université, Paris, France; 4 Department of Social and Health Care, Psychosocial Services Adolescent Outpatient Clinic Kauppakatu 14, Lahti, Finland; 5 Department of Child and Adolescent Psychiatry and Psychotherapy, Central Institute of Mental Health, Medical Faculty Mannheim, Heidelberg University, Mannheim, Germany; 6 Department of Psychiatry, CHU Sainte-Justine Hospital, University of Montréal, Montreal, QC Canada; 7 Division of Psychiatry, University College of London, London, UK; 8 Department of Child and Adolescent Psychiatry Psychosomatics and Psychotherapy, Campus CharitéMitte, Charité-Universitätsmedizin Berlin, Charitéplatz 1, Berlin, Germany; 9 Department of Child and Adolescent Psychiatry and Psychotherapy, University Medical Center, von-Siebold-Str. 5, 37075 Göttingen, Germany; 10 Discipline of Psychiatry, School of Medicine and Trinity College Institute of Neuroscience, Trinity College Dublin, Dublin, Ireland; 11 Centre for Population Neuroscience and Precision Medicine (PONS), Institute of Psychiatry, Psychology & Neuroscience, Social, Genetic and Developmental Psychiatry Centre, King’s College London, London, UK; 12 Institute of Cognitive and Clinical Neuroscience, Central Institute of Mental Health, Medical Faculty Mannheim, Heidelberg University, Square J5, Mannheim, Germany; 13 Department of Psychology, School of Social Sciences, University of Mannheim, 68131 Mannheim, Germany; 14 NeuroSpin, CEA, Université Paris-Saclay, F-91191 Gif-sur-Yvette, France; 15 Departments of Psychiatry and Psychology, University of Vermont, Burlington, VT 05405 USA; 16 Sir Peter Mansfield Imaging Centre School of Physics and Astronomy, University of Nottingham, University Park, Nottingham, UK; 17 Department of Psychiatry and Psychotherapy CCM, Charité—Universitätsmedizin Berlin, corporate member of Freie Universität Berlin, Humboldt-Universität zu Berlin, and Berlin Institute of Health, Berlin, Germany; 18 Physikalisch-Technische Bundesanstalt (PTB), Braunschweig and Berlin, Germany; 19 Institute of Medical Psychology and Medical Sociology, University Medical Center Schleswig Holstein, Kiel University, Kiel, Germany; 20 Department of Psychiatry and Neuroscience, Faculty of Medicine, CHU Sainte-Justine Research Center, Population Neuroscience Laboratory, University of Montreal, Montreal, QC Canada; 21 Section of Systems Neuroscience, Medical Faculty, Technische Universität Dresden, Dresden, Germany; 22 School of Psychology and Global Brain Health Institute, Trinity College Dublin, Dublin, Ireland; 23 Centre for Population Neuroscience and Stratified Medicine (PONS), ISTBI, Fudan University Shanghai and Department of Psychiatry and Neuroscience, Charité University Medicine, Berlin, Germany; 24 Department of Psychiatry, EPS Barthélémy Durand, Etampes, France; 25 INSERM, U1299, Ecole Normale Supérieure Paris-Saclay, Gif-sur-Yvette, France; 26 Physikalisch-Technische Bundesanstalt, Dresden, Germany; 27 Central Institute of Mental Health, Mannheim, Germany; 28 Commissariat à l’Energie Atomique, Saclay, France; 29 King’s College London, London, UK; 30 Trinity College Dublin, Dublin, Ireland; 31 INSERM U1299, Gif-sur-Yvette, France; 32 University of Hamburg, Hamburg, Germany; 33 Physikalisch-Technische Bundesanstalt, Berlin, Germany; 34 Montreal University, Montreal, QC Canada; 35 University of Vermont, Burlington, VT USA; 36 University of Nottingham, Nottingham, UK; 37 Charité Universitätsmedizin Berlin, Berlin, Germany; 38 Technische Universität Dresden, Dresden, Germany; 39 Assistance Publique Hôpitaux de Paris APHP, Paris, France; 40 University of Montreal, Montreal, QC Canada; 41 University of Toronto, Toronto, ON Canada; 42 University of Göttingen, Göttingen, Germany; 43 Delosis, London, UK; 44 University of Helsinki, Helsinki, Finland

**Keywords:** Predictive markers, Psychiatric disorders

## Abstract

Recent longitudinal studies in youth have reported MRI correlates of prospective anxiety symptoms during adolescence, a vulnerable period for the onset of anxiety disorders. However, their predictive value has not been established. Individual prediction through machine-learning algorithms might help bridge the gap to clinical relevance. A voting classifier with Random Forest, Support Vector Machine and Logistic Regression algorithms was used to evaluate the predictive pertinence of gray matter volumes of interest and psychometric scores in the detection of prospective clinical anxiety. Participants with clinical anxiety at age 18–23 (*N* = 156) were investigated at age 14 along with healthy controls (*N* = 424). Shapley values were extracted for in-depth interpretation of feature importance. Prospective prediction of pooled anxiety disorders relied mostly on psychometric features and achieved moderate performance (area under the receiver operating curve = 0.68), while generalized anxiety disorder (GAD) prediction achieved similar performance. MRI regional volumes did not improve the prediction performance of prospective pooled anxiety disorders with respect to psychometric features alone, but they improved the prediction performance of GAD, with the caudate and pallidum volumes being among the most contributing features. To conclude, in non-anxious 14 year old adolescents, future clinical anxiety onset 4–8 years later could be individually predicted. Psychometric features such as neuroticism, hopelessness and emotional symptoms were the main contributors to pooled anxiety disorders prediction. Neuroanatomical data, such as caudate and pallidum volume, proved valuable for GAD and should be included in prospective clinical anxiety prediction in adolescents.

## Introduction

Anxiety disorders have been reported to have a high impact on the global burden of disease [1]. Anxiety disorders are the most prevalent psychiatric condition in adolescence, impacting nearly one in three individuals [2, 3]. The average age of onset predates 15 years old for social anxiety disorder and specific phobia, whereas panic disorder and generalized anxiety disorder tend to emerge slightly later in life [4]. Moreover, anxiety disorders can remain unstable in adolescence, before consolidating further in young adulthood. Therefore, detecting individuals at elevated risk of developing clinical anxiety is crucial.

Many sociodemographic, psychosocial, as well as physical and mental health factors have been reported as risk factors of generalized anxiety disorder and panic disorder [5]. Personality scores such as neuroticism and anxiety sensitivity have also been established as pre-existing risk factors for future anxiety disorders [6, 7]. Early-life anxious temperament is also a strong risk factor for anxiety disorders later in life [8]. Furthermore, neuroimaging data, jointly with psychometric and clinical data, show promise to identify at-risk populations [9].

In adolescent patients with anxiety disorders, cross-sectional differences in gray matter volume have been reported using magnetic resonance imaging (MRI) in the amygdala, hippocampus, insula, cingulate cortex, ventromedial prefrontal cortex (vmPFC), and temporal gyri [10]. Additionally, the striatum was highlighted as a critical subcortical region of interest for the onset of anxiety disorders in adolescence [11]. A model of anxious temperament has also been shown to involve the central amygdala, orbitofrontal cortex, bed nucleus of the stria terminalis (BNST) and periaqueductal gray in young primates [12, 13].

However, only few longitudinal studies have investigated neurostructural correlates of anxiety in adolescents from a developmental perspective ([14], see [15] for a review). It was reported that amygdala volume measured up to three times at 2-year intervals in non-clinical participants between age 4 and 18 was longitudinally and positively associated with anxio-depressive symptoms [16]. Two studies were based on regions of interest (ROIs) but did not include limbic structures. One reported that larger pituitary volume at age 12–13 preceded an increase in anxiety symptoms 2–3 years later [17]. The other found that larger right middle temporal gyrus cortical thickness in non-clinical participants aged 13 to 20 predicted symptoms of generalized anxiety disorder 2 years later [18].

Nonetheless, statistical association does not necessarily translate into cross-sectional classification or prospective prediction, the second and third implying an ability to generalize findings to new, unseen data [19]. In the last decade, research in psychiatry has progressively incorporated machine-learning approaches in an effort to bridge the gap between diagnostic or prognostic markers detected at group level, and clinical relevance. Machine-learning techniques have shown promise in single-subject patient classification using neuroimaging data, and there has been a recent effort to use larger samples and multisite data to overcome inherent limitations of such analyses [20]. Few studies in adults have attempted single-participant classification of clinical anxiety using neuroimaging data, with limited sample sizes, heterogenous performance metrics and non-prospective designs. In adults, two studies investigated classification of social anxiety disorder with very small clinical sample sizes (*N*_patients_ = 14 and 20, with accuracies of 0.845 and 0.825 respectively) [21, 22], one other study did so with moderately larger sample size (*N*_patients_ = 47, area under the curve = 0.72) [23], and another investigated spider phobia classification (*N*_patients_ = 59, accuracy ranging from 0.62 to 0.88) [24]. In each study, classification performance tended not to rely on a few select structures of the fear circuitry or other networks, but rather relied on diffuse predictors across the brain. In adolescents, to our knowledge, only one prospective prediction of anxiety has been attempted, in which orbitofrontal cortex volume and orbitofrontal-amygdala functional connectivity in a dot-probe task at age 7–17 were found to be predictive of social anxiety score a year later in a healthy adolescent sample (support vector regression *r*_(predicted_, _observed)_ = 0.301) [25].

Therefore, the first aim of the current study was to predict prospective clinical anxiety at the individual level at ages 18 and/or 23, both pooled and disorder-specific, based on gray matter volumes as well as psychometric features such as neuroticism and anxiety sensitivity scores at age 14. The second aim was to assess the respective contributions of both gray matter volumes and psychometric feature categories to the prediction performance. These analyses were conducted under the a priori hypotheses that gray matter volumes in subcortical and frontomedial regions might have, and that psychometric features would have, a predictive value for the onset of anxiety in adolescence.

## Methods

### Dataset and sample description

All data originated from the IMAGEN database [26] that includes neuroimaging data collected in community adolescents at age 14, as well as several questionnaires evaluating mental disorders, emotional functioning and alcohol and substance consumption. Written informed consent was obtained from all participants and their legal guardians.

Diagnostic data were collected at baseline, at age 18–19 (first follow-up, FU1), and age 22–23 (FU2) using the DAWBA (Development And Well-Being Assessment), a computerized self-report assessment that generates DSM-IV and ICD-10 diagnoses [27]. These diagnoses were subsequently evaluated by trained clinicians, as previously described [28]. Alcohol and cannabis consumption were respectively evaluated using the AUDIT (Alcohol Use Disorders Identification Test) and the ESPAD (European School survey Project on Alcohol and other Drugs) [29, 30]. Other clinical assessments included negative thinking, anxiety sensitivity subscales from the Substance Use Risk Profile Scale (SURPS) [31]; emotional symptoms score in the Strength and Difficulties Questionnaire (SDQ) [32]; autonomy, accidents, distress, family, and relocation subscales from the Life Events Questionnaire (LEQ) (adapted from [33]); neuroticism, and extraversion subscales from the revised NEO Personality Inventory (NEO-FFI) [34]; novelty-seeking as measured by the revised Temperament and Character Inventory (TCI-R) [35]. A more detailed description of the questionnaires is presented in Supplementary methods.

**Fig. 1:**
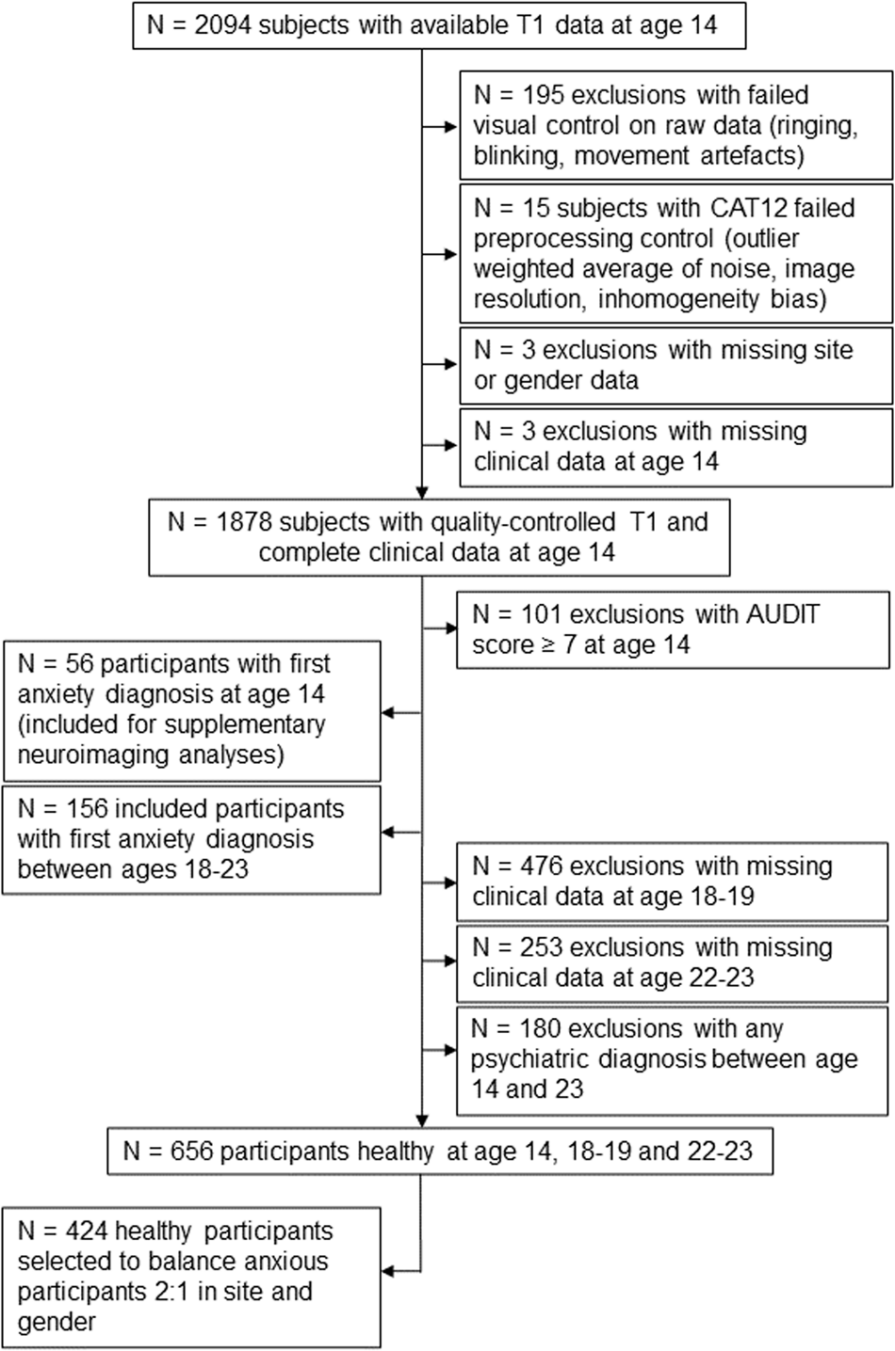
Inclusion flowchart. AUDIT Alcohol Use Disorder Identification Test.

Participants with T1 data at baseline were assessed for eligibility in our analyses. Visual quality control was conducted for each MRI T1 scan and participants with excessive noise, motion artefacts or abnormal brain anatomy were excluded. Participants with AUDIT scores equal to or greater than 7 at baseline were excluded, as alcohol disorder may interfere with brain structure development [36] (inclusion flowchart in Fig. 1).

Participants with DAWBA anxiety diagnoses of generalized anxiety disorder (GAD), social anxiety disorder (SAD), specific phobia (SpP), panic disorder (PD), agoraphobia (AG) and other anxiety (OA) at baseline, FU1 or FU2 were included. They were subdivided between those who had at least one anxiety diagnosis at baseline (BLA) at age 14 (*N* = 56, only used in Supplementary neuroimaging group analyses), and future anxiety-onset participants, whose first anxiety diagnosis was reported at either FU1 or FU2 (future anxiety, FUA). FUA participants were then allocated to 5 mutually exclusive groups. Those who had only one anxiety disorder diagnosis at 18–23 (one stable diagnosis at both FU1 and FU2, or one diagnosis at either FU1 or FU2) were split into GAD, SAD, SpP and PD/AG diagnostic groups, while participants with multiple anxiety disorders (mAD) at any timepoint at ages 18 and/or 23 (i.e., two or more distinct anxiety diagnoses, simultaneous or not) were allocated to a mAD group (see Supplementary Tables 1 and 2 for details about the FUA or BLA sample respectively). PD and AG were combined because they are highly comorbid disorders [37], and our sample size did not allow the investigation of standalone agoraphobia. Participants that had missing DAWBA data at FU1 or FU2 but did have one anxiety diagnosis at the other follow-up timepoint (FU2 or FU1 respectively) were included, as this latter criterion was sufficient for allocation to the FUA (32 participants) or BLA (24 participants) groups. A total of *N* = 156 FUA participants were available for prediction analyses.

Eligible controls were typical adolescents with no DAWBA diagnosis at baseline, FU1 and FU2. Participants with incomplete DAWBA data at any timepoint were excluded from eligible controls. Then, we randomly selected controls amongst eligible participants to balance scanning acquisition sites and gender with participants with anxiety disorders by a 2:1 ratio.

### MRI acquisition and preprocessing

All scans were obtained on 3T scanners (Siemens, Philips, General Electrics) across the 8 IMAGEN European sites, based on an ADNI-MPRAGE standardized acquisition sequence (sagittal plane, 2.3 ms repetition time, 2.93 ms echo time, 8° flip angle, 256 × 256 × 160 matrix, voxel size 1.1 × 1.1 × 1.1 mm) [26]. MRI data were preprocessed with the CAT12 toolbox version 12.6 (http://www.neuro.uni-jena.de/cat/) in SPM12 (Statistical Parametric Mapping, https://www.fil.ion.ucl.ac.uk/spm/) in Matlab (https://fr.mathworks.com). T1-weighted images were segmented, normalized, and modulated. They were smoothed with a statistical 8 mm Full-Width Half-Maximum Gaussian filter (final voxel size: 1.5 × 1.5 × 1.5 mm).

CAT12 provides TIV (Total Intracranial Volume) measures and calculates image quality ratings (noise, inhomogeneity bias, image resolution, and a weighted average rating of these measures, obtained with a root mean square equation to accentuate the impact of a mediocre measurement). The weighted average rating was examined, and the worse scoring participants (D and C-) at baseline were excluded from all analyses.

### Machine-learning prediction

#### Feature extraction

Extraction was conducted with SPM12. ROIs classically involved in clinical anxiety were extracted from the AAL atlas [38] and combined from the left and right hemispheres with the WFU_PickAtlas toolbox (https://www.nitrc.org/projects/wfu_pickatlas/). ROIs included the amygdala, hippocampus, parahippocampal gyrus, mid- and anterior cingulate cortex, gyrus rectus, medial orbitofrontal cortex, putamen, pallidum, caudate nucleus, thalamus, insula, as well as the periaqueductal gray (PAG) from a 6 mm sphere centered on *x* = 0, *y* = −29, *z* = −12 [39], and the BNST [40]. Gray matter volumes for each ROI were extracted from preprocessed scans with the MarsBar toolbox [41] with no additional scaling, for a total of 14 neuroimaging features at age 14.

IMAGEN questionnaire subscales relevant to anxiety phenomenology (including novelty-seeking, emotional symptoms, autonomy, accidents, distress, family, relocation, hopelessness, anxiety sensitivity, alcohol consumption, neuroticism, and extraversion) were selected a priori. Age at baseline (in days) was also included to account for its potential interactions with other features, resulting in a total of 13 psychometric features at age 14.

#### Machine-learning pipeline

Classifications were conducted with scikit-learn 0.24.2 (https://scikit-learn.org/dev/versions.html) in Python. A majority voting algorithm between Logistic Regression (LR), Support Vector Machine (SVM) and Random Forest (RF) classifiers was used.

Three separate binary class prediction analyses were conducted with baseline neuroimaging and psychometric data. The first analysis was the prediction of any FUA (*N* = 156) vs. healthy controls (*N* = 424). The second analysis was the prediction of FUA GAD diagnosis (*N* = 42) vs. healthy controls, and the third prediction of FUA mAD (*N* = 42) vs. healthy controls. Only the GAD and mAD groups had more than 30 FUA participants. Thus, no other specific diagnosis group could be explored. As the data were moderately imbalanced between FUA participants and healthy controls, additional functions from imbalanced-learn 0.8.0 [42] were used. The FUA SpP, SAD and PD/Ag groups all included *N* < 30 subjects and could not be explored separately (*N* = 25, *N* = 25, and *N* = 22 respectively). The three above-mentioned predictions were first conducted with the 27 features together, then only with the 13 psychometric features, and only with the 14 regional gray matter volume features, to evaluate their respective contributions.

A leave-3-groups-out cross-validation strategy was used: in each cross-validation fold, 5 acquisition sites were chosen as training data and the remaining 3 sites as testing data, such that no two participants from the same site could be in both the training and testing sets (see Supplementary methods for distribution of participants across sites). All possible splits of the 8 sites resulted in 56 cross-validation folds in total, and in each fold a nested stratified 5-fold hyperparameter optimization to maximize area under the receiver operating curve (AUROC) was conducted. Inside each nested fold, missing psychometric data (0.02% of questionnaire scores in the whole sample, including FUA and healthy controls, *N* = 580) were imputed with the feature median, then data were scaled and resampled with a combination of over- and under-sampling (synthetic minority oversampling technique and edited nearest neighbors cleaning with default parameters) so that both groups would have equal size [43]. The analysis pipeline and reported metrics (i.e., 10-fold cross-validation, nested preprocessing to avoid data leakage, AUROC reported as a performance metric insensitive to relative class frequencies) were chosen according to recommended practices [19]. Mean performance metrics over the 56 folds are reported in the results section.

The “liblinear” library was set as the solver parameter of the LR classifier, and the class weight parameter was set to “balanced” for all three classifiers. Optimized hyperparameters included the number of maximum iterations, penalty and C from the LR classifier, the gamma and C from the SVM classifier, and the maximum depth and maximum number of features from the RM classifier. Scikit-learn default values were used for all remaining classifier parameters.

To examine each feature contribution to individual predictions more closely, we also used the recent Shapley additive explanation (SHAP) module, version 0.39.0 [44]. SHAP uses a game theoretic approach to assign an importance value to each feature for an individual prediction and allows visualization of the contribution of each feature value to its final classification for each participant.

## Results

### Sample characteristics

FUA participants did not differ from healthy controls for age, gender and TIV at baseline, but they had significantly higher AUDIT, ESPAD, neuroticism, anxiety sensitivity and emotional symptoms scores (see Supplementary Table 1). Additionally, significantly higher neuroticism and emotional symptoms scores were detected in FUA mAD participants compared to FUA participants with only one diagnosed disorder (*p* value = 5.5e−3 and 2.3e−2 respectively).

### Machine-learning diagnostic predictions

**Fig. 2:**
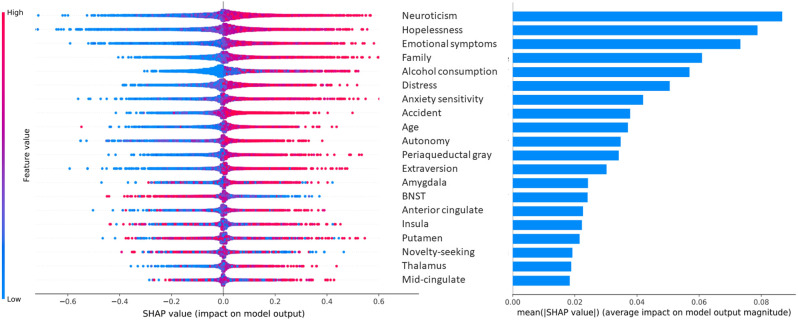
SHAP values and importance of features at age 14 in the prediction of any future anxiety (*N* = 156) vs. healthy control (*N* = 424). Each dot represents an individual in a given cross-validation iteration. Positive Shapley values indicate contribution of a feature value in favor of the positive class (future anxiety) prediction, negative Shapley values are in favor of the negative class (healthy control) prediction. Larger absolute Shapley values indicate larger impact on the model output. The 20 most contributing features are shown. BNST bed nucleus of the stria terminalis.

Trained classifiers for all cross-validation iterations of all analyses are available online (https://osf.io/pdmrv/). Prediction of any future anxiety disorder vs. healthy control class resulted in an AUROC = 0.68 (standard deviation (SD) = 0.03) (Table 1). Features that most differentiated between classes included neuroticism, hopelessness, emotional symptoms and family events (Fig. 2). Higher values were interpreted by the trained classifier as contributing to clinical anxiety outcome classification, rather than to the healthy control class. Greater bilateral BNST volume supported healthy control classification outcome.

**Table 1:** Mean performance metrics of both pooled and disorder-specific future anxiety vs. healthy controls predictions.

Classification metric	AUROC (SD)			Balanced accuracy (SD)			Sensitivity (SD)			Specificity (SD)		
Feature set	N + P	N	P	N + P	N	P	N + P	N	P	N + P	N	P
Any future anxiety (*N* = 156) vs. HC (*N* = 424)	0.68 (0.03)	0.52 (0.04)	0.69 (0.03)	0.60 (0.04)	≤0.5^a^ (0.03)	0.63 (0.04)	0.81 (0.13)	0.79 (0.17)	0.67 (0.15)	0.38 (0.15)	0.21 (0.17)	0.58 (0.12)
Future GAD (*N* = 42) vs. HC (*N* = 424)	0.69 (0.07)	0.63 (0.06)	0.62 (0.08)	0.62 (0.08)	0.59 (0.04)	0.57 (0.07)	0.53 (0.23)	0.49 (0.21)	0.38 (0.26)	0.71 (0.12)	0.69 (0.16)	0.76 (0.16)
Future mAD (*N* = 42) vs. HC (*N* = 424)	0.71 (0.06)	≤0.5^a^ (0.06)	0.74 (0.05)	0.65 (0.06)	≤0.5^a^ (0.05)	0.67 (0.07)	0.63 (0.20)	0.44 (0.24)	0.59 (0.12)	0.67 (0.14)	0.51 (0.22)	0.75 (0.10)

*HC* healthy controls, *GAD* generalized anxiety disorder, *mAD* multiple anxiety disorder, *AUROC* area under the receiver operating curve, *N* analysis conducted using neurostructural (regional gray matter volumes) features, *P* analysis conducted using psychometric features.

^a^Equal to or below chance level.

**Fig. 3:**
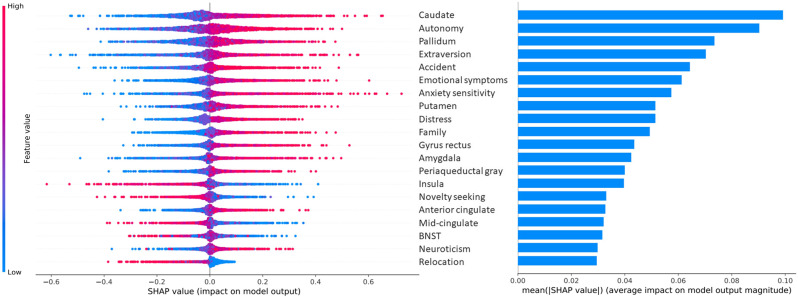
SHAP values and importance of features at age 14 in the prediction of future generalized anxiety disorder (*N* = 42) vs. healthy control (*N* = 424). Each dot represents an individual in a given cross-validation iteration. Positive Shapley values indicate contribution of a feature value in favor of the positive class (future generalized anxiety) prediction, negative Shapley values are in favor of the negative class (healthy control) prediction. Larger absolute Shapley values indicate larger impact on the model output. The 20 most contributing features are shown. BNST bed nucleus of the stria terminalis.

Prediction of FUA GAD resulted in an AUROC = 0.69 (SD = 0.07). Most contributing features included bilateral caudate volume, autonomy, bilateral pallidum volume, extraversion, accident score, emotional symptoms and anxiety sensitivity, with higher values supporting FUA GAD outcome classification (Fig. 3). Larger bilateral insula, BNST and mid-cingulate volumes, as well as higher novelty-seeking and relocation scores, contributed to healthy control classification outcome.

**Fig. 4:**
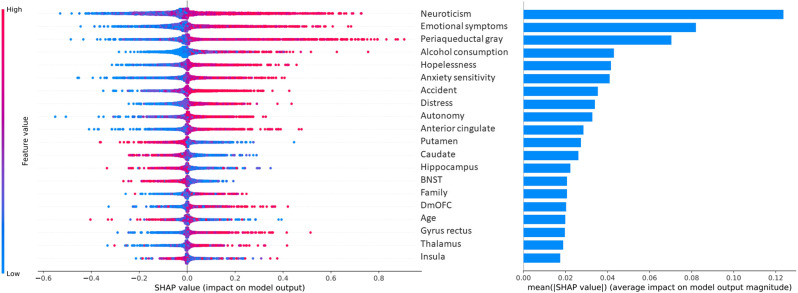
SHAP values and importance of features at age 14 in the prediction of future multiple anxiety diagnoses (*N* = 42) vs. healthy control (*N* = 424). Each dot represents an individual in a given cross-validation iteration. Positive Shapley values indicate contribution of a feature value in favor of the positive class (future multiple anxiety diagnoses) prediction, negative Shapley values are in favor of the negative class (healthy control) prediction. Larger absolute Shapley values indicate larger impact on the model output. BNST bed nucleus of the stria terminalis, DmOFC dorsomedial orbitofrontal cortex. The 20 most contributing features are shown.

Prediction of FUA mAD resulted in an AUROC = 0.71 (SD = 0.06). Most impacting features included neuroticism, emotional symptoms and PAG volume, with higher values supporting FUA mAD outcome classification (Fig. 4). Larger bilateral putamen, caudate, BNST, hippocampus and insula volumes, as well as age, generally supported healthy control classification outcome. A prediction of FUA GAD vs. FUA mAD is presented in Supplementary Fig. 1.

### Contribution of neuroimaging features

Predicting any future anxiety vs. healthy control based on gray matter volumes alone resulted in an AUROC = 0.52 (SD = 0.04), and the same prediction based only on psychometric features resulted in an AUROC = 0.69 (SD = 0.03).

Predicting FUA GAD vs. healthy control based on gray matter volumes alone resulted in an AUROC = 0.63 (SD = 0.06) and predicting based only on psychometric features resulted in an AUROC = 0.62 (SD = 0.08).

Predicting FUA mAD vs. healthy control based on gray matter volumes alone resulted in an AUROC ≤ 0.50 (SD = 0.06) and predicting based only on psychometric features resulted in an AUROC = 0.74 (SD = 0.05).

## Discussion

This is the first report of anxiety onset prediction in European adolescents at age 18–23, using regional gray matter volumes and clinical features obtained at age 14. The predictive value of gray matter volumes alone for future anxiety disorders was also investigated. Prediction performance was above chance level when all future anxiety diagnoses were pooled together, with the most contributing features being neuroticism and hopelessness scores. No contribution of neuroimaging features in classical regions of interest for anxiety was found in the prediction of *pooled* anxiety disorders. However, bilateral caudate and pallidum volumes at age 14 were major contributors to the specific prediction of pure GAD at age 18–23, with larger volumes in both regions indicating future GAD diagnosis. Additionally, prediction of future multiple anxiety disorders (mAD) across late adolescence involved a larger periacqueductal gray volume.

### Predictive features for GAD, mAD and pooled diagnoses

As there is no pre-existing prediction study of future anxiety in adolescents, our prediction performance can only be put in perspective with two recent predictions of prospective depression and bipolar disorder (AUROC = 0.72 and 0.76 respectively) [45, 46]. Indeed, using psychometric and neuroimaging features together, our prediction performance of pooled anxiety diagnoses was close (AUROC = 0.68), while our follow-up period was longer (8 years vs. 5 years follow-up in both studies). However, the performance should still be improved for prospective anxiety individual prediction to be clinically useful.

Regional gray matter features showed no incremental contribution to the prediction of pooled anxiety with respect to psychometric data alone in our analysis. Additionally, regional gray matter features alone were poorly predictive of pooled diagnoses or mAD (AUROC = 0.52 and ≤0.50 respectively). One possible explanation for the lack of incremental accuracy could be the limited number of features used in our analysis (discussed in the limitations). Indeed, several predictive studies of anxiety using neuroimaging data report diffuse contributions to the prediction performance across the brain [21–24]. An alternative explanation could be that, although many neuroimaging similarities have been reported in clinical anxiety across diagnoses, heterogeneities remain between anxiety disorders [47, 48]. Herein, larger caudate and pallidum volumes were predictive of pure GAD, but reduced volumes of the same regions were predictive of mAD, and these volumes were among the most contributing features for a GAD vs. mAD prediction (Supplementary Fig. 1). This heterogeneity might explain why regional gray matter volumes alone were better predictors of a specific diagnosis like GAD (AUROC = 0.63).

The striatum is frequently overlooked in the anxiety literature in comparison to the amygdala, anterior insula, bed nucleus of the stria terminalis, hippocampus and vmPFC. However, its potential importance, particularly in the emergence of anxiety during adolescence, has been highlighted in the past [11]. Our findings are in line with that vision, and caudate and pallidum volumes were also significantly larger in future GAD participants compared with healthy controls in a voxel-based morphometry group analysis (Supplementary Fig. 2b and Supplementary Table 3b).

Additionally, the periacqueductal gray was significantly larger in baseline mAD participants (see Supplementary Fig. 2a and Supplementary Table 3a). The periaqueductal gray is involved in defensive behavior and pain processing but has also been implicated in fear, anxiety and anxious temperament for more than two decades despite being often eclipsed by other nodes such as the amygdala, vmPFC, and BNST [13, 49]. However, its predictive value to prospective mAD was only moderate in comparison to the psychometric questionnaires, when all 27 features were used.

Neuroticism was the most predictive feature of future anxiety in the pooled anxiety sample. Still, although neuroticism was the psychometric subscale most relevant to anxiety in our dataset, it is not a plain measure of clinical severity and anxiety symptoms. Rather, neuroticism is a personality trait strongly associated with experiencing intense negative emotions and with internalizing disorders [50, 51]. Herein, participants with mAD at age 18–23 had higher neuroticism and emotional symptoms mean scores at age 14 (before anxiety onset) than participants who were going to develop only one disorder. Our findings further confirm that neuroticism plays a role in anxiety onset during adolescence, perhaps denoting broad vulnerability to multiple anxiety disorders.

Following decades of MRI and fMRI group analyses, machine-learning individual predictions with neurofunctional and neuroanatomical markers show promise as one of the next steps toward targeted monitoring and treatment of psychiatric disorders [20, 52]. The above-mentioned features most important to our prediction could contribute to the identification of a teenage population at risk of developing anxiety disorders in the following years, and to an early detection of disease.

### Strengths

Generalizability is a traditional issue in individual prediction, some concerns of which were addressed here. First, the IMAGEN cohort includes data from community adolescents collected from multiple acquisition sites across Europe and has an 8-years follow-up time that covers a window of vulnerability from teenage to young adulthood, leading to a good ecological validity. To our knowledge, IMAGEN is the largest neuroimaging cohort currently available spanning a period from puberty to early adulthood. Secondly, our machine-learning analysis included a state-of-the-art pipeline with appropriate nested cross-validation procedures to circumvent for the limited sample size and data imbalance. Our cross-validation strategy in particular was preferred over a more traditional K-fold one (see Supplementary results) to capitalize on the multicentric nature of the IMAGEN dataset in an effort to improve the generalizability of prediction performance. Finally, SHAP was used to maximize interpretability.

### Study limitations

The sample sizes for anxiety disorder groups were the main limitation as a consequence of the long follow-up interval, and made separate prediction for social anxiety disorder, panic disorder with and without agoraphobia as well as specific phobia, impossible. In order to reduce the risk of overfitting with the limited sample sizes, we did not use nested feature selection (to avoid the risk of the algorithm selecting features based on very few GAD or mAD participants) and restricted our a priori selection to a small number of features [53]. Regional gray matter features, which are of specific interest during adolescence, were chosen over neurofunctional data in our analysis and measures from both hemispheres were combined, but future studies are also encouraged to explore neurofunctional predictors of anxiety onset whenever possible. One other possible limitation may be that IMAGEN participants were recruited in the general population and not through any clinical institution. It was, however, a necessary design to investigate *prospective* psychiatric disorders.

The IMAGEN cohort was not designed for the investigation of clinical anxiety, particularly not at age 14, and, as such, does not include targeted and specific clinical constructs assessing overall and diagnosis-specific anxious severity, such as the LSAS for social anxiety [54]. One could hypothesize that using questionnaires specific to clinical anxiety as features would improve the performance of both pooled and separate diagnosis prediction. Moreover, although the DAWBA, used to determine diagnostic status in the database, is a clinically valid diagnostic instrument [27], it does not optimally assess the exact time of symptom onset.

Finally, it must be noted that gender and site were not used as predictive features, despite the well-known gender difference in anxiety disorders [55], as they were the initial balancing criteria between participants with anxiety and healthy groups.

## Conclusion

The present study substantiates that clinical anxiety could be prospectively and individually predicted in teenagers using a multisite approach, albeit with moderate performance. Prediction performance showed that easily collected psychometric features, mainly neuroticism, hopelessness, and emotional symptoms at 14, greatly contributed to the prediction of pooled anxiety diagnoses. Thus, the present findings further support the idea that self-screening of these clinical features in teenagers could contribute to the early detection of anxiety disorders. Additionally, specific anxiety diagnosis prediction relied on some regional gray matter features such as striatal volumes, warranting further investigation of their involvement in developmental anxiety.

## Supplementary information

Supplemental material

## Data Availability

The code used in prediction analyses can be made available upon request.
