# Supplementary material for: Anxiety onset in adolescents: a machine-learning prediction
Source: Mol Psychiatry. 2022 Dec 8;28(2):639–46. doi: 10.1038/s41380-022-01840-z (PMC9908534; doi:10.1038/s41380-022-01840-z)
Supplement: Supplementary file 1 — Supplemental material [file 41380_2022_1840_MOESM1_ESM.docx]

Supplementary methods

Neuroimaging group-level analysis

All analyses were conducted with SPM12 and CAT12. Gray matter volume analyses were conducted within an anatomical mask encompassing bilateral regions classically involved in clinical anxiety, built from the AAL atlas with the WFU_PickAtlas toolbox (https://www.nitrc.org/projects/wfu_pickatlas/). The gray matter volume (GMV) mask included the amygdala, hippocampus, parahippocampal gyrus, mid- and anterior cingulate cortex, gyrus rectus, medial orbitofrontal cortex, putamen, pallidum, caudate nucleus, thalamus, insula, midbrain (the latter taken from the TD Lobes atlas) and BNST (1) regions of interest (ROIs).

Cross-sectional one-way ANOVA analyses were conducted with a group factor over the GM images at baseline with age, gender, IMAGEN acquisition sites, TIV, DAWBA depressive comorbidity, AUDIT score, and ESPAD cannabis consumption score as covariates of no interest. A six-level group factor was used for FUA participants (SpP, SAD, PD/AG, GAD, mAD or controls) and pairwise contrasts were examined. As only mAD had N>20 BLA participants, a two-level group factor was used (mAD or controls).

Results were obtained using the TFCE toolbox (<http://www.neuro.uni-jena.de/tfce/>) for non-parametric permutation-based statistics on each contrast, with a family-wise error-corrected p_FWE_ ≤ 0.05 threshold and more than 10 voxels. TFCE parameters were set as 5000 permutations with the Smith method, with default weights H (height) and E (extent) (H = 2, E = 0.5 for GMV).  Anatomical location of significant clusters was determined with the AAL atlas and manually verified with MRIcron (<https://www.nitrc.org/projects/mricron>) for GMV.

Psychometric questionnaire features

The life-event questionnaire (LEQ) assesses a range of potentially stressful activities or experiences in adolescents, and the family, autonomy, distress, accident and relocation frequencies subscales (lifetime frequency score for distress, family and accident, past year score for autonomy and relocation) were included as predictive features. The revised temperament and character inventory (TCI-R) measures excitability, disorderliness, impulsivity and extravagance for a sum score of novelty seeking which was used as a feature. The strength and difficulties questionnaire (SDQ) measures conduct problems, peer problems, prosocial behaviour, hyperactivity, as well as emotional symptoms which was included in our analysis. The substance use risk profile scale (SURPS) assesses dimensions of sensation-seeking, impulsivity, hopelessness and anxiety sensitivity, and the last two were included as features. Finally, the revised NEO personality inventory (NEO-FFI is a well-known personality assessment based on the five-factor model, and measures openness, agreeableness, conscientiousness, extraversion and neuroticism subscales, with the last two being included in our analysis. While age is not a specific predictor of anxiety, age (in days) was included in our analysis to account for its potential interactions with other features, which could also provide predictive value.

Diagnosis stability

Among the 89 FUA participants that had a first anxiety diagnosis at FU1, 9 had the same anxiety disorder(s) diagnosed at FU2, 2 had the same diagnosis and one additional anxiety disorder diagnosed at FU2, 7 had a different anxiety diagnosis at FU2, 51 had no anxiety diagnosis at FU2, and 20 did not have clinical data at FU2.

FUA participants distribution across sites

In the FUA group, data were collected in Berlin for N = 15 participants, in Dresden for N = 16 participants, in Dublin for N = 17 participants, in Hamburg for N = 17 participants, in London for N = 33 participants, in Mannheim for N = 6 participants, in Nottingham for N = 29 participants, and in Paris for N = 23 participants.

Alternative cross-validation strategy

A more traditional 10-fold stratified cross-validation repeated 10 times was also explored for completeness, with the nested optimization and all other classifier parameters kept identical.

Supplementary Results

Sample characteristics for BLA participants

At baseline, no significant difference was observed between BLA participants and healthy controls for age, gender, TIV, AUDIT or ESPAD score. BLA participants had significantly higher neuroticism (NEO), anxiety sensitivity (SURPS) and emotional symptoms (SDQ) scores.

Additional machine-learning prediction

With a 10-fold stratified cross-validation, the prediction of any anxiety disorder vs. healthy control resulted in an AUROC = 0.69 (SD = 0.06), with sensitivity = 0.82 (SD = 0.11) and specificity = 0.42 (SD = 0.13) and balanced accuracy = 0.62 (SD = 0.06).

Prediction of GAD vs. healthy control resulted in an AUROC = 0.71 (SD = 0.11), with sensitivity = 0.63 (SD = 0.28), specificity = 0.66 (SD = 0.12) and balanced accuracy = 0.65 (SD = 0.12).

Prediction of mAD vs. healthy control resulted in an AUROC = 0.77 (SD = 0.13) with sensitivity = 0.67 (SD = 0.28), specificity = 0.69 (SD = 0.12) and balanced accuracy = 0.68 (SD = 0.12).

Neuroimaging group analyses

Early adolescence anxiety onset

At age 14, a larger volume in the periaqueductal gray (p_FWE_ = 0.039) was detected in participants with early onset of mAD compared to healthy controls (see Supplementary Fig. 1A and Supplementary Table 2A for details). Contrasting all pooled participants with anxiety disorders (SpP, SoP, PD/Ag, GAD and mAD together) with first onset at age 14 with healthy controls yielded no significant differences.

Late adolescence/early adulthood anxiety onset

At age 14, a larger volume in the caudate nucleus was detected bilaterally (p_FWE_ = 0.017 for the left and 0.032 for the right hemisphere) in participants with future GAD compared to controls (see Supplementary Fig. 1B and Supplementary Table 2B for details). Contrasting all FUA participants together (SpP, SoP, PD/Ag, GAD and mAD) with healthy controls yielded no significant differences, and neither did separate SpP, Soph, PD/Ag and mAD analyses.

Supplementary DiScussion

Using participants from the IMAGEN cohort, we found a significantly larger gray matter volume in the bilateral caudate of non-anxious participants at age 14 that were going to develop a generalized anxiety disorder (GAD) in the next 8 years.

Additional limits of the neuroimaging group analysis

The limited patient sample size in our analysis led to the use of covariates of no interest, such as depressive comorbidity, rather than exclusion. It must be noted that, as the database included no medication nor psychotherapy data at age 14, their potential confounding effects could not be considered.

| Group | SpP | SAD | PD/Ag | GAD | Multiple anxiety diagnoses ^a^ | Total FUA | HC | p-value FUA vs. HC |
| --- | --- | --- | --- | --- | --- | --- | --- | --- |
| N participants | 25 | 25 | 22 | 42 | 42 | 156 | 424 |  |
| N major or other depression diagnoses at age 14 | 2 | 2 | 2 | 0 | 1 | 7 |  |  |
| Gender (m/f) | 3/22 | 10/15 | 5/17 | 12/30 | 10/32 | 40/116 | 131/293 | n.s. |
| Age (years)  (SD) | 14.6  (0.5) | 14.4  (0.4) | 14.4  (0.3) | 14.4  (0.3) | 14.4  (0.5) | 14.4  (0.4) | 14.4  (0.4) | n.s. |
| AUDIT score  (SD) | 1.2  (1.7) | 0.9  (1.5) | 2.0  (1.9) | 1.4  (2.0) | 1.4  (1.5) | 1.4  (1.8) | 0.9  (1.4) | 1.0e-3* |
| ESPAD-year score  (SD) | 0.3  (1.1) | 0.1  (0.4) | 0 | 0.1  (0.4) | 0.1  (0.5) | 0.1  (0.6) | 0.1  (0.4) | 2.9e-2* |
| Neuroticism NEO score  (SD) | 26.7  (8.0) | 28.0  (7.2) | 25.0  (8.5) | 24.9  (6.6) | 29.5  (7.7) | 27.0  (7.7) | 22.6  (6.7) | 5.4e-10* |
| Anxiety sensitivity SURPS score  (SD) | 11.5  (2.9) | 12.6  (2.0) | 11.2  (2.5) | 12.1  (2.5) | 12.6  (2.5) | 12.1  (2.5) | 11.2  (2.1) | 2.5e-5* |
| SDQ emotional score  (SD) | 4.0  (2.4) | 3.7  (2.5) | 2.9  (2.5) | 3.2  (1.8) | 4.4  (2.3) | 3.7  (2.3) | 2.5  (1.8) | 1.1e-8* |
| Transversal TIV (mm^3^)  (SD) | 1 461.1  (132.5) | 1 460.2  (144.6) | 1 428.9  (118.9) | 1 498.9  (133.5) | 1 441.8  (140.4) | 1 461.5  (135.7) | 1 453.0  (133.5) | n.s. |

**Supplementary Table 1: Sample description at age 14 of participants with future anxiety**

SpP: Specific phobia; SAD: social anxiety disorder; PD/Ag: panic disorder and/or agoraphobia; GAD: generalized anxiety disorder; HC: healthy controls; anxiety diagnosed at age 18-19 or age 22-23 follow-ups.

* p <0.05; Kruskal-Wallis test for continuous variables, Chi^2^ test for categorical variables.

^a^ Group included participants meeting criteria for at least two diagnoses (SpP, SoPh, PD/Ag, GAD, or other clinical anxiety (OA)), simultaneously or not, at any of the BL, FU1 or FU2 timepoints: 42 participants had a first anxiety onset at FU1/FU2 (9 SpP, 24 SoPh, 16 PD/Ag, 25 GAD and 12 OA current or future diagnoses).

**Supplementary Table 2: Sample description at age 14 of participants with current anxiety**

| Group | SpP | SAD | PD/Ag | GAD | Multiple anxiety diagnoses ^a^ | Total BLA | HC | p-value BLA vs. HC |
| --- | --- | --- | --- | --- | --- | --- | --- | --- |
| N participants | 8 | 10 | 8 | 7 | 23 | 56 | 424 |  |
| N depression diagnoses at age 14 | 1 | 4 | 4 | 4 | 3 | 16 |  |  |
| Gender (m/f) | 2/6 | 3/7 | 2/6 | 1/6 | 4/19 | 12/44 | 131/293 | n.s. |
| Age (years)  (SD) | 14.3  (0.3) | 14.3  (0.4) | 14.3  (0.2) | 14.2  (0.3) | 14.3  (0.4) | 14.3  (0.3) | 14.4  (0.4) | n.s. |
| AUDIT score  (SD) | 0.8  (1.2) | 0.8  (1.4) | 0.8  (1.0) | 1.9  (1.7) | 1.3  (1.7) | 1.1  (1.5) | 0.9  (1.4) | n.s. |
| ESPAD-year score  (SD) | 0 | 0 | 0 | 0 | 0.0  (0.2) | 0.0  (0.1) | 0.1  (0.4) | n.s. |
| Neuroticism NEO score  (SD) | 27.1  (11.0) | 32.1  (8.1) | 35.0  (7.1) | 28.0  (7.5) | 32.4  (5.9) | 31.4  (7.7) | 22.6  (6.7) | 1.1e-13* |
| Anxiety sensitivity SURPS score  (SD) | 11.6  (2.9) | 12.1  (1.5) | 11.8  (2.4) | 12.1  (2.1) | 12.9  (2.8) | 12.3  (2.5) | 11.2  (2.1) | 4.3e-3* |
| SDQ emotional score  (SD) | 4.1  (2.2) | 5.6  (2.5) | 5.8  (1.9) | 5.0  (1.9) | 5.6  (1.6) | 5.3  (1.9) | 2.5  (1.8) | 3.6e-18* |
| Transversal TIV (mm^3^)  (SD) | 1 463.1  (128.9) | 1 542.2  (156.3) | 1 442.1  (95.1) | 1 374.5  (148.5) | 1 435.3  (123.5) | 1 451.7  (134.8) | 1 453.0  (133.5) | n.s. |

SpP: Specific phobia; SAD: social anxiety disorder; PD/Ag: panic disorder and/or agoraphobia; GAD: generalized anxiety disorder; HC: healthy controls; BLA: anxiety diagnosed at age 14.

* p <0.05; Kruskal-Wallis test for continuous variables, Chi^2^ test for categorical variables.

^a^ Group included participants meeting criteria for at least two diagnoses (SpP, SoPh, PD/Ag, GAD, or other clinical anxiety(OA)), simultaneously or not : 23 participants had a first anxiety onset at baseline (10 SpP, 18 SoPh, 10 PD/Ag, 14 GAD and 4 OA current or future diagnoses).

**Supplementary Figure 1 : SHAP values of features and importance at age 14 in the generalized anxiety (N=42) vs. multiple anxiety (N=42) prediction**


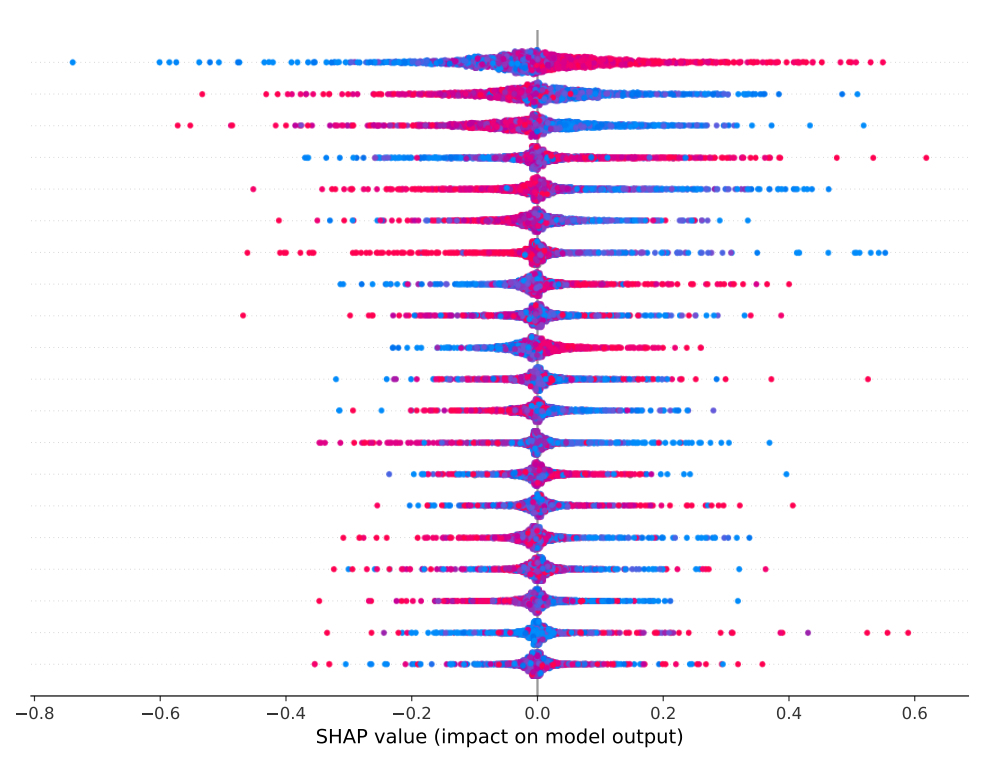

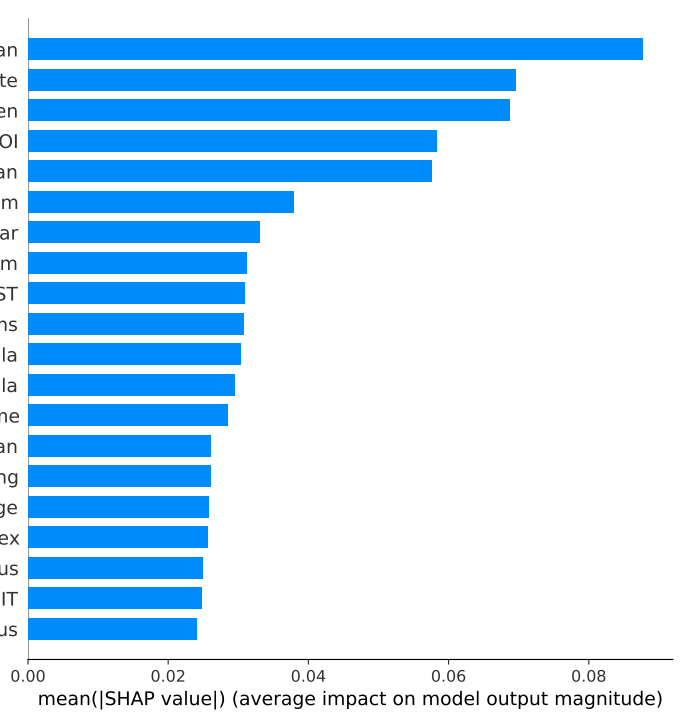


Neuroticism

Caudate

Putamen

Periaqueductal gray

Extraversion

Pallidum

Autonomy

Mid-cingulate

BNST

Emotional symptoms

Insula

Amygdala

Family

Hopelessness

Novelty-seeking

Age

DmOFC

Gyrus rectus

Alcohol consumption

Thalamus


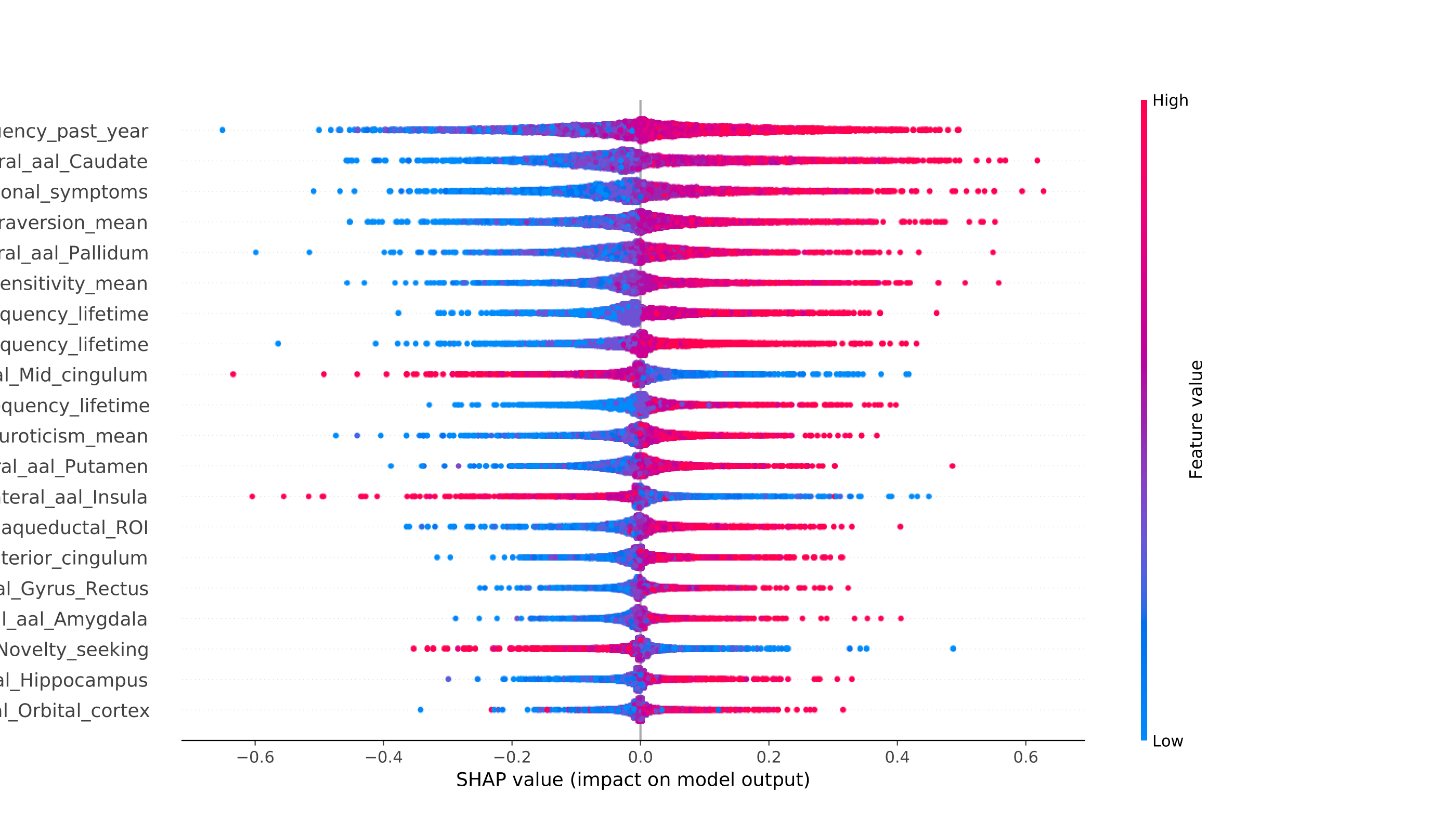


Positive Shapley values indicate contribution of a feature value in favour of the positive class (multiple anxiety) prediction, negative Shapley values are in favour of the negative class (generalized anxiety) prediction. BNST: bed nucleus of the stria terminalis; DmOFC: dorsomedial prefrontal cortex. The 20 most contributing features are shown.

**Supplementary Figure 2: Gray matter volume differences at age 14 between participants with current or future anxiety and healthy controls**


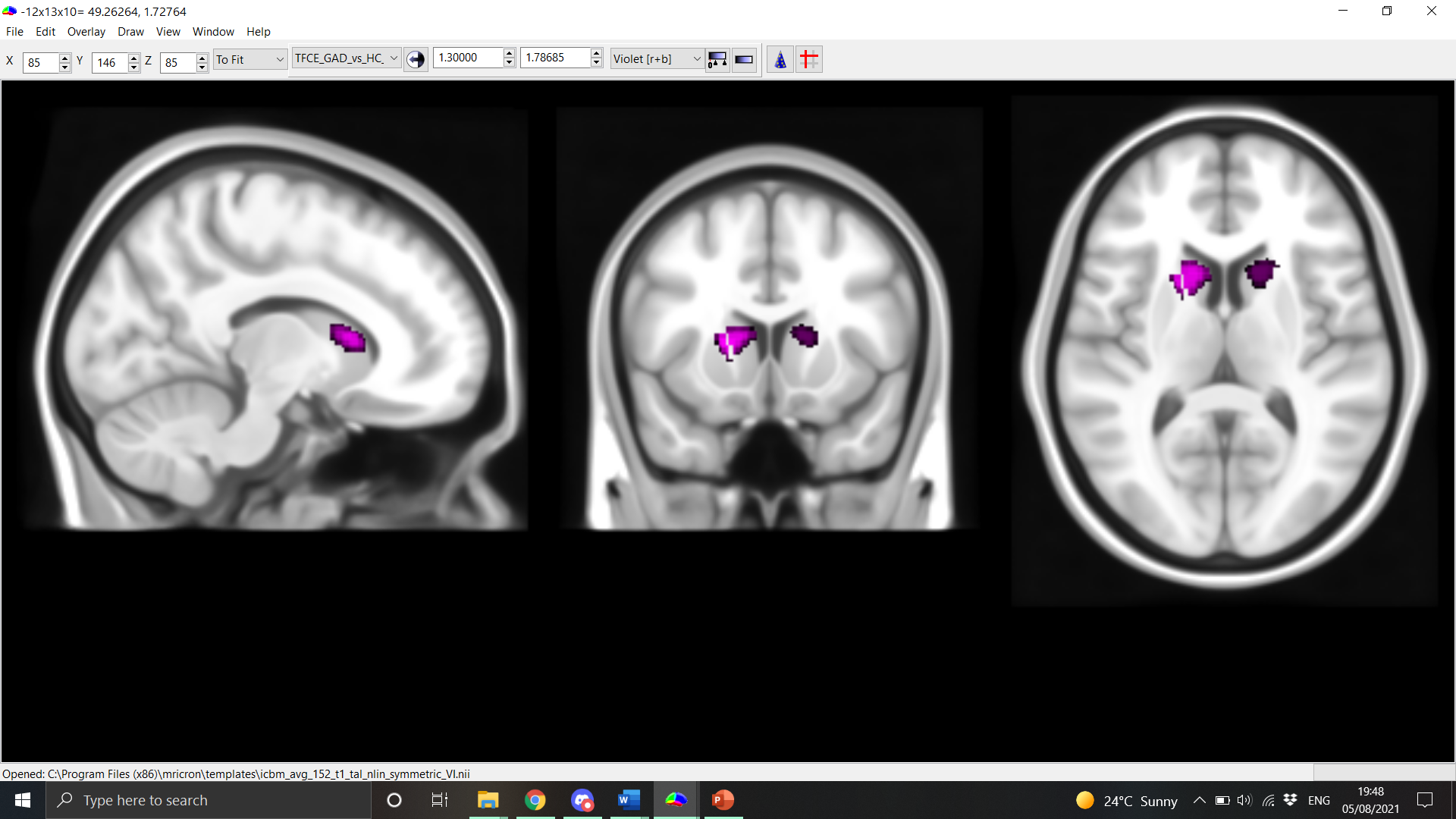


A.

B.


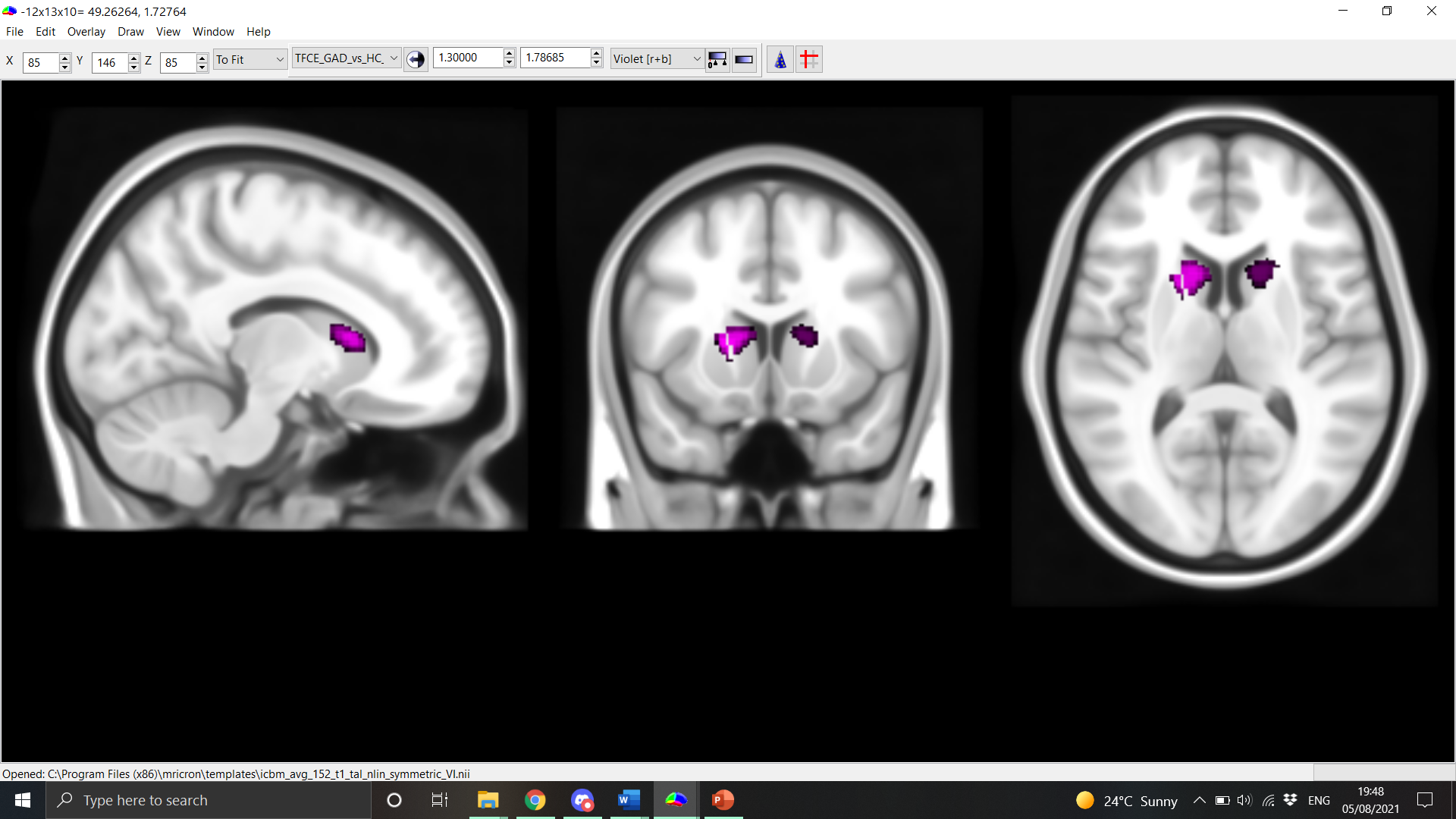

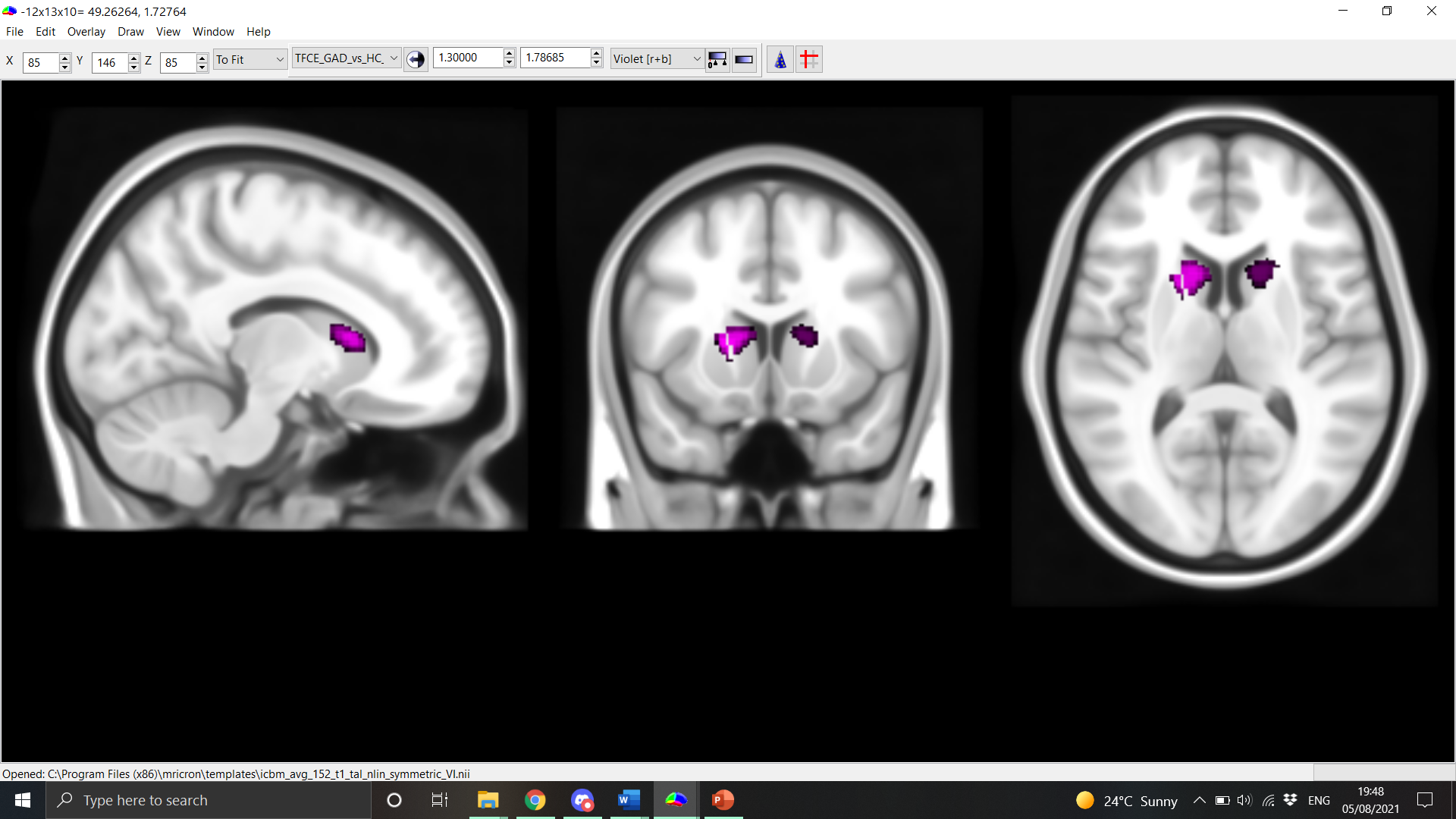


X = 0

Y = -29

Z = -16

X = 12

Y = 13

Z = 10


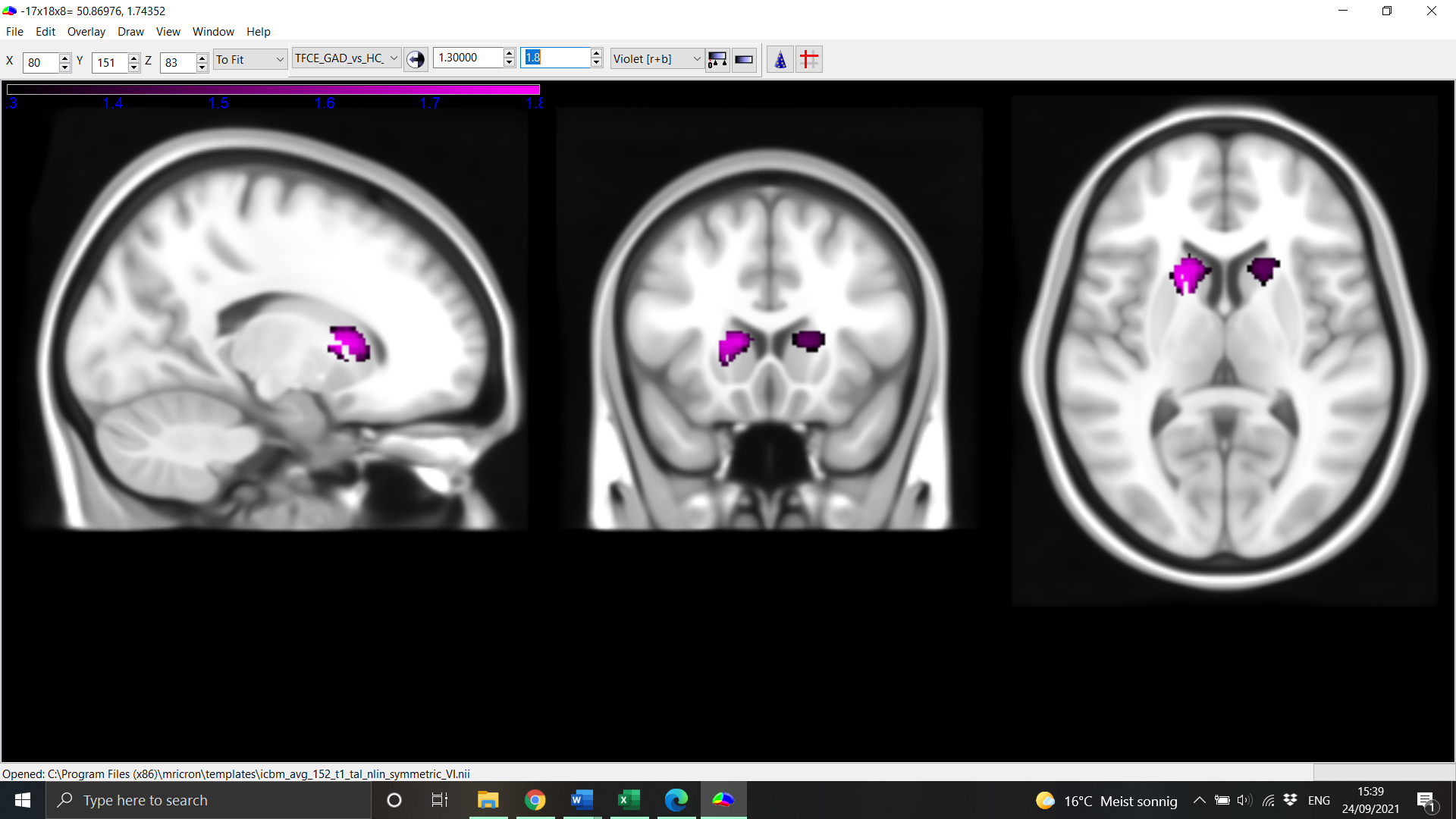


- Log(p)

1.3

1.4

1.5

1.6

1.7


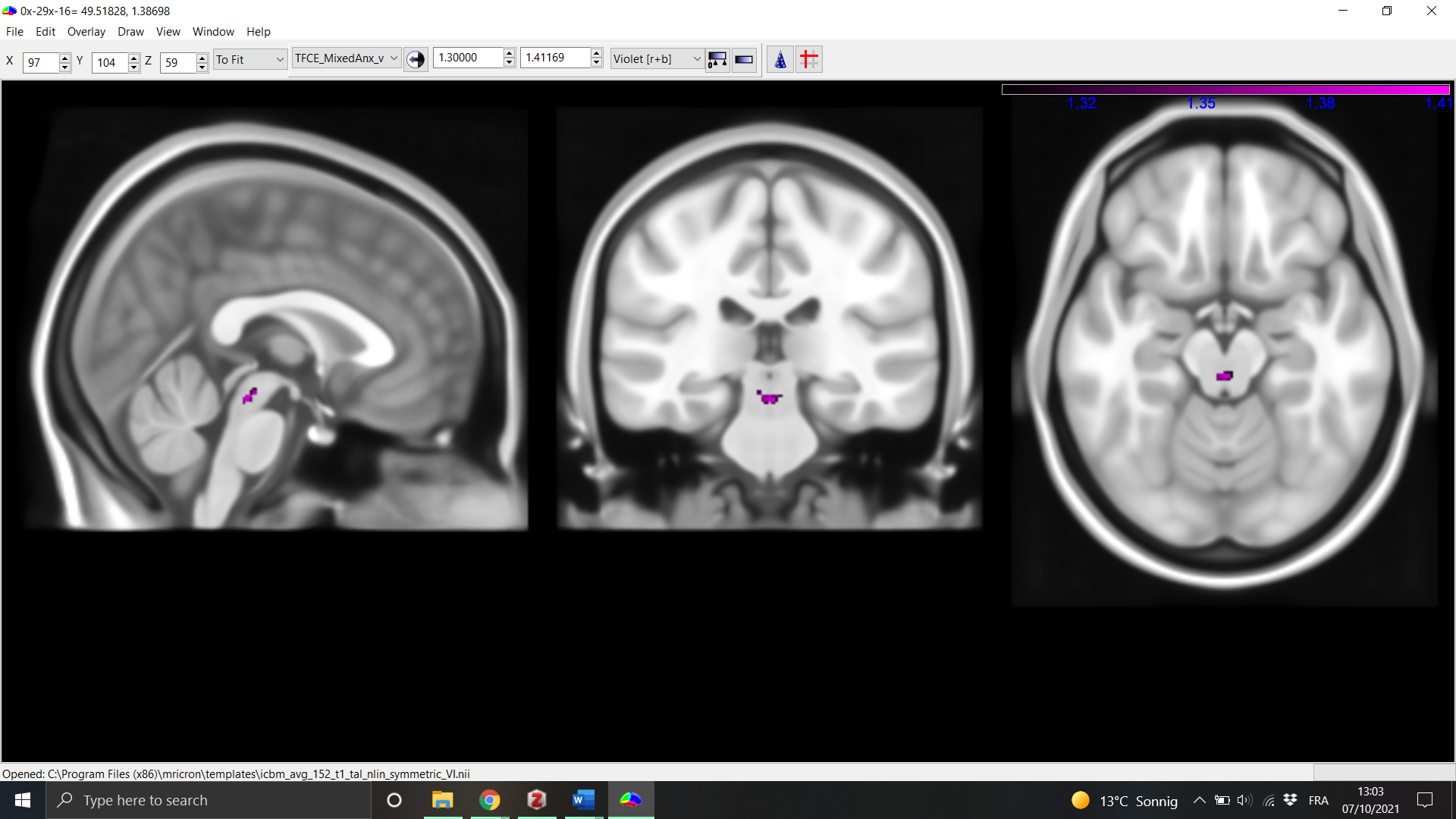

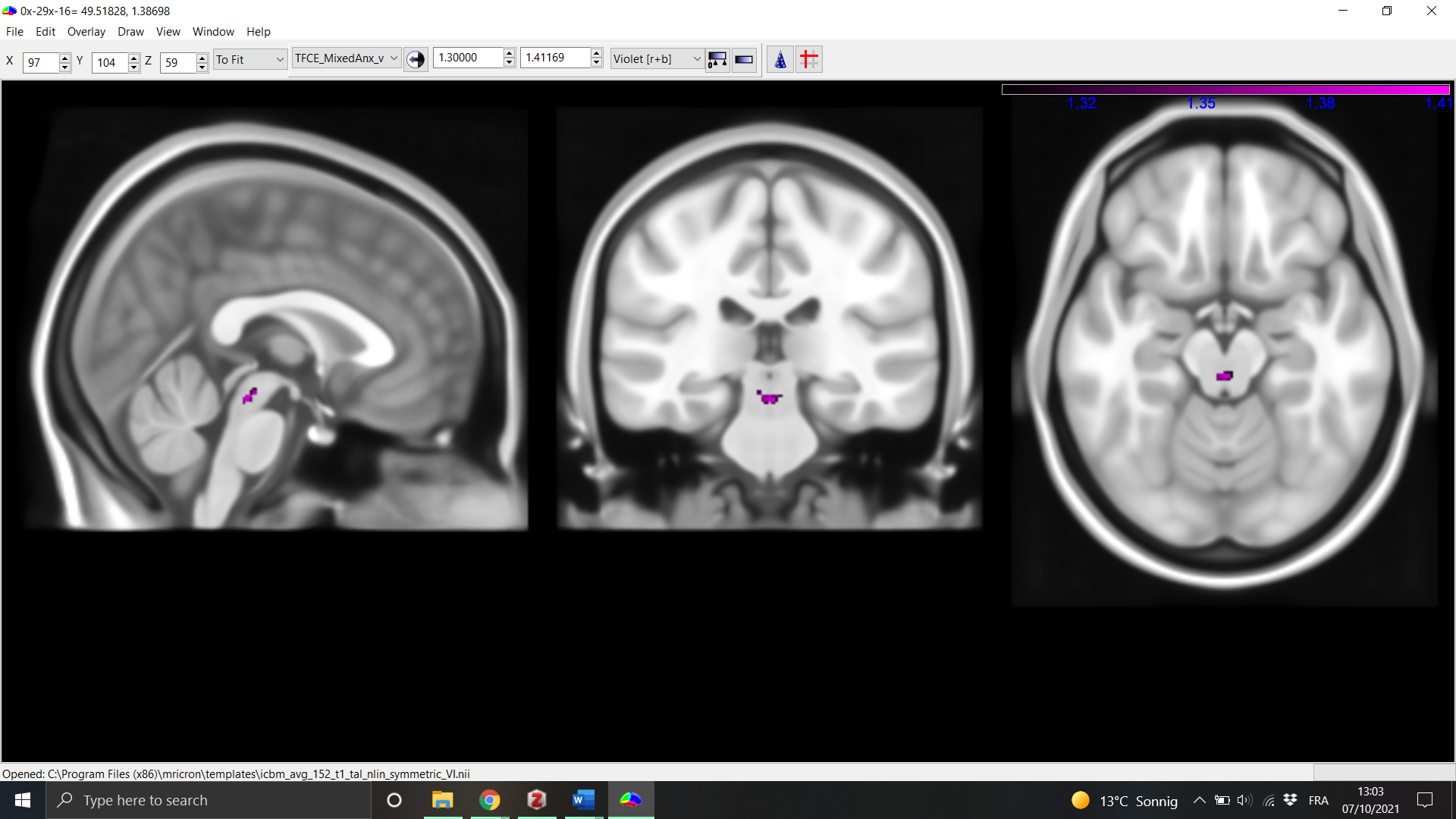

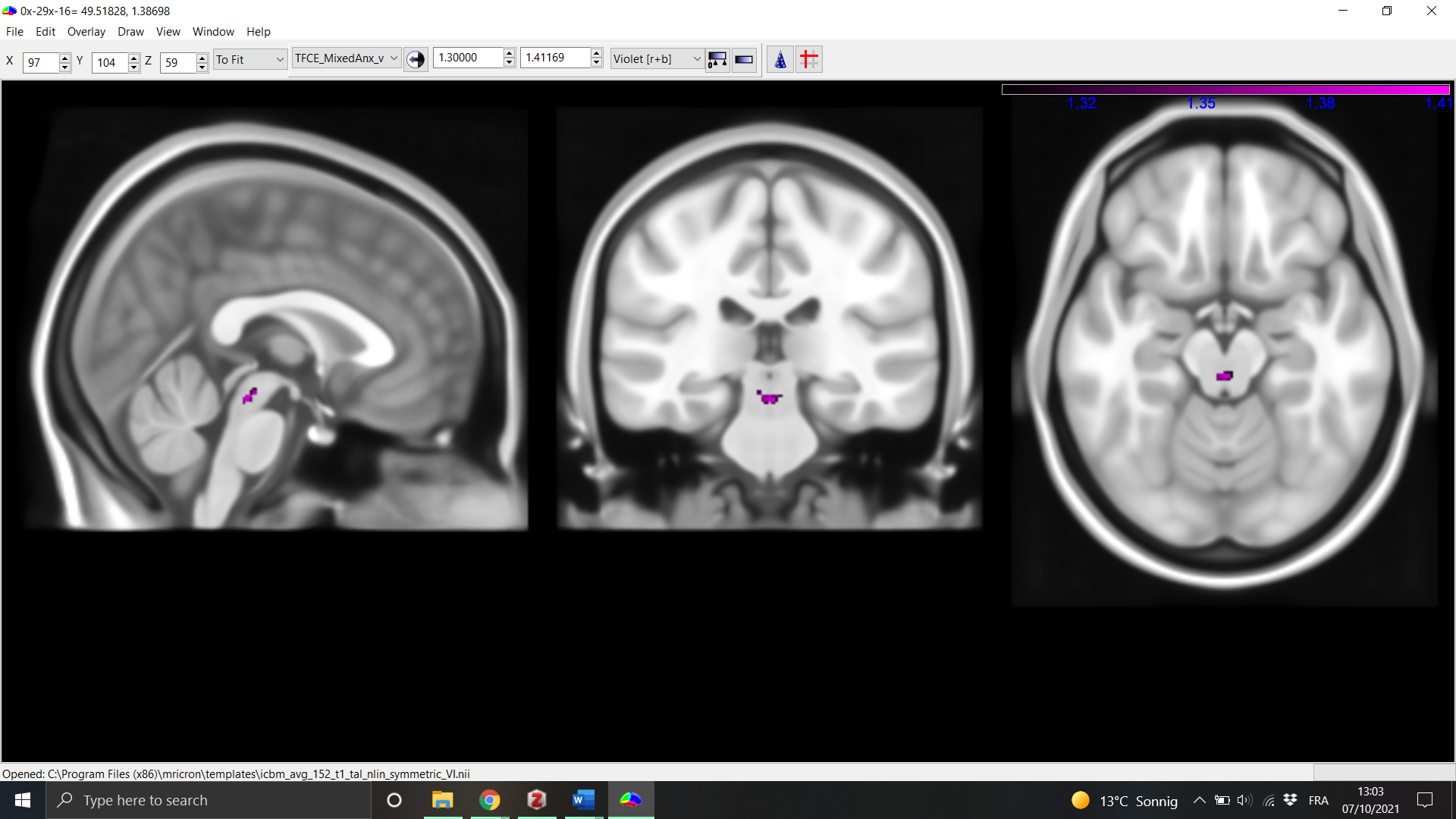

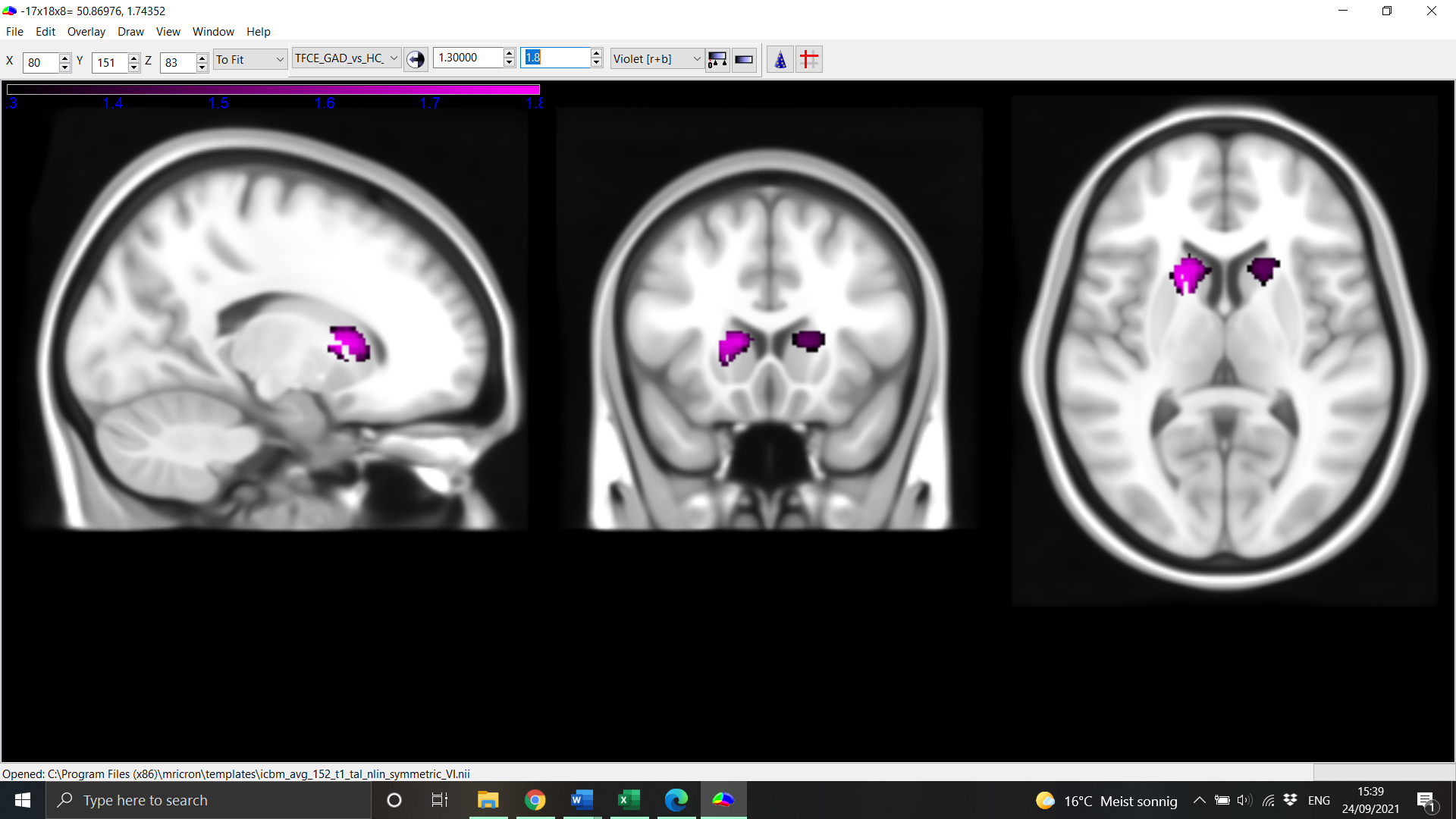


1.8

- Log(p)

1.41

1.35

1.38

1.32

1.3

1.8

1.8

1.8

1.8

A: Brain regions larger at age 14 in participants with current first onset of multiple anxiety disorders (N = 23) than in healthy controls (N = 424); B: Brain regions larger at age 14 in participants with future onset of generalized anxiety disorder (N = 42) than in healthy controls (N = 424).

p_FWE_ ≤ 0.05, clusters > 10 voxels reported.

- Log(p)

**Supplementary Table 3: Gray matter volume differences at age 14 between participants with current or future anxiety and healthy controls**

| Contrast | | | Combined cluster-peak | | | | | |
| --- | --- | --- | --- | --- | --- | --- | --- | --- |
|  | k | Region | TFCE | p_uncorr_ | p_FWE_ | MNI coordinates | | |
|  |  |  |  |  |  | x | y | z |
| A. **BLA** |  |  |  |  |  |  |  |  |
| Mixed anxiety > controls | 37 | Periacqueductal gray | 497.12 | 6.0e-4 | 0.039 | 0 | -27 | -15 |
|  |  |  |  |  |  |  |  |  |
| Controls > mixed anxiety |  | n.s. |  |  |  |  |  |  |
|  |  |  |  |  |  |  |  |  |
| B. **FUA** |  |  |  |  |  |  |  |  |
| GAD > controls | 560 | L. Putamen | 608.17 | 2.0e-4 | 0.016 | -18 | 12 | 8 |
|  |  | L. Caudate | 602.27 | 2.0e-4 | 0.017 | -15 | 16 | 9 |
|  | 261 | R. Caudate | 521.66 | 4.0e-4 | 0.032 | 16 | 16 | 9 |
|  |  |  |  |  |  |  |  |  |
| Controls > GAD |  | n.s. |  |  |  |  |  |  |
|  |  |  |  |  |  |  |  |  |

A: Volume differences between participants with onset of mAD at age 14 (N=23) and healthy controls (N=424); B: Volume differences at age 14 between participants with future onset of GAD (N=42) and healthy controls (N=424).

BLA: anxiety diagnosed at age 14; FUA: anxiety diagnosed at age 18-19 or age 22-23 follow-ups; GAD: generalized anxiety disorder; mAD: multiple anxiety disorders, simultaneous or not.

P<0.05 FWE-corr. Clusters >10 voxels reported. No significant volume difference was found between the FUA GAD and FUA mAD groups at age 14.

references

1. Neudorfer C, Germann J, Elias GJB, Gramer R, Boutet A, Lozano AM. A high-resolution in vivo magnetic resonance imaging atlas of the human hypothalamic region. Sci Data. 2020 Sep 15;7(1):305.
